# National Resident Matching Program Performance Among US MD and DO Seniors in the Early Single Accreditation Graduate Medical Education Era

**DOI:** 10.7759/cureus.17319

**Published:** 2021-08-20

**Authors:** Michael W Kortz, Austin Vegas, Sean P Moore, Edwin McCray, Monica C Mureb, Jacob E Bernstein, Joshua May, Brandon Bishop, Mitchell Frydenlund, John R Dobson

**Affiliations:** 1 Neurosurgery, University of Colorado School of Medicine, Aurora, USA; 2 Osteopathic Medicine, Kansas City University College of Osteopathic Medicine, Kansas City, USA; 3 Osteopathic Medicine, Campbell University School of Osteopathic Medicine, Buies Creek, USA; 4 Neurosurgery, New York Medical College Westchester Medical Center, Westchester, USA; 5 Neurosurgery, Riverside University Health System Medical Center, Moreno Valley, USA; 6 Pathology, Kansas City University College of Osteopathic Medicine, Kansas City, USA

**Keywords:** national resident matching program, residency application, graduate medical education, medical students, allopathic, osteopathic, medical school

## Abstract

Introduction: As of the 2020 National Resident Matching Program (NRMP), nearly all applicants are evaluated together for graduate medical education (GME) candidacy. We set out to characterize US MD and DO Senior residency match performance in the single-accreditation GME era.

Methods: A retrospective study was conducted in 2021 utilizing data collected from the 2018 and 2020 NRMP Charting Outcomes in the Match publications aggregated and subdivided into three groups based on competitiveness: low (LC), moderate (MC), and high (HC). Nonparametric analysis was performed using Chi square or Fisher exact tests if counts were less than five. Significance was determined at p < 0.05.

Results: A total of 46,853 candidates were included, with 36,194 (77.3%) US MD and 10,659 (22.7%) DO Seniors. Match rates for US DO Seniors were lower than US MD Seniors across all competitiveness strata (p < 0.0001). Research item production, national licensing examination scores, and mean number of contiguous programs ranked were lower for matched US DO Seniors compared to matched US MD Seniors, with significant differences depending on competitiveness group.

Conclusions: With recent changes to GME and its application process, understanding how various groups compare will be increasingly important. US DO Seniors have lower first-rank match rates for all specialty competitiveness levels. This may be due to lower research output or nuanced specialty selection. This study could aid GME stakeholders to more effectively allocate resources and better prepare residency candidates.

## Introduction

There are two degree pathways toward physicianship in the United States: Doctor of Medicine (MD) and Doctor of Osteopathic Medicine (DO) [1-2]. While both can be licensed to practice in all medical specialties, their training has historically remained separate. There were two organizations that accredited graduate medical education (GME) training programs before 2020: the Accreditation Council for Graduate Medical Education (ACGME) and the American Osteopathic Association (AOA) [3]. Although DO candidates could apply to ACGME-accredited programs, MD candidates could not apply to AOA-accredited ones. In 2014, the ACGME, AOA, and American Association of Osteopathic Medical Colleges (AACOM) announced a partnership that would bring all residency programs under a new single-accreditation GME system starting in 2016 and becoming fully integrated in 2020 [4]. The system aims to homogenize GME, promote collaboration, and improve training nationwide [5].

To gain an ACGME-accredited residency training position in all specialties except urology and ophthalmology, candidates must participate in the National Resident Matching Program (NRMP), i.e. “The Match” [6]. All candidates must excel in their academic work, national licensing examinations (NLEs), research, and extracurriculars; but there is a growing emphasis on holistic evaluation [7-10]. Considering that a majority of US DO medical schools do not have affiliated hospital systems, osteopathic medical students’ access to mentorship and research resources may be limited compared to their allopathic colleagues [11]. This is especially important as the first exams of the United States Medical Licensing Examination (USMLE) and Comprehensive Osteopathic Medical Licensing Examination (COMLEX) transition to a pass/fail grading system in 2022, which had previously served as proxy equalizers for candidates from less connected or prestigious institutions [12-13].

There remains a paucity of studies investigating how US MD and DO Senior applicant profiles compare with regard to various factors. Additionally, many subspecialties have few US DO applicants, prohibiting definitive analysis. To rectify the limitations of previous research, we set out to determine how these two groups have fared in the single accreditation GME system, adjusted for specialty competitiveness. This study may delineate how key factors in US MD and DO Senior applicant profiles cause match rate discrepancies, expand the conversation of potential bias against candidates, and elucidate opportunities for institutions to better support applicants.

## Materials and methods

A retrospective population-based study was conducted to characterize and compare US MD and DO Senior NRMP performance in the early single accreditation GME era, adjusted for specialty competitiveness. Data were collected from the 2018 and 2020 NRMP Charting Outcomes in the Match (COM) publications, which reports data from US MD and DO Seniors who apply to residency and rank individual programs through the AAMC’s Electronic Residency Application Service (ERAS) [14-17]. Individual, de-identified data were requested from the NRMP but was not available. After data were collected, it was subsequently aggregated and coded for analysis. Otolaryngology did not participate in the 2018 NRMP and thus data were not available for this year. All other programs and specialties, regardless of historical MD/DO match rates or individual program specifics, were included in our data sets.

Only matched US MD and DO Seniors were compared to limit confounding by non-reportable factors. International medical graduates and previously graduated candidates were excluded. NRMP specialties were initially analyzed individually, but there were several specialties with too few DO candidates to accommodate statistically significant analysis. To increase the sample power, specialties were stratified into three competitiveness cohorts: low-competitiveness (LC), moderate-competitiveness (MC), and high-competitiveness (HC). These stratifications were created by using the ratio of US senior applications who matched successfully into their preferred specialty to the number of positions available in said specialty based on the calculated Match Quality Score which has been published previously [7, 11] (Table 1).

**Table 1:** Specialty competitiveness stratification by match quality score [7]. LC, low competitiveness; MC, moderate competitiveness; HC, high competitiveness

LC (n=20,570)	MC (n=21,171)	HC (n=5,112)
Family medicine	Anesthesiology	Dermatology
Internal medicine	Diagnostic radiology	Neurosurgery
Neurology	Emergency medicine	Orthopedic surgery
Pathology	General surgery	Otorhinolaryngology
Physical medicine and rehabilitation	Internal medicine and pediatrics	Plastic surgery
Psychiatry	Pediatrics	Vascular surgery
	Obstetrics and gynecology	Interventional radiology
	Child neurology	

Data were then collected on match rates, number of candidates’ contiguous programs ranked, USMLE Step 1 and 2 CK (Clinical Knowledge) scores, and research output. The NRMP determines match rate by the simple proportions of candidates who placed into their first-choice specialty against all applicants in that specialty. Research output is defined in two ways: number of projects (i.e., "research experiences”) and number of abstracts, presentations, and publications (i.e., “research items”). A two-level contingency was then created (those with <5 or ≥5 research items or experiences) for comparison. NLE performance was determined by USMLE Step 1 and 2 CK score. Very few MD candidates take COMLEX and a significant portion of DO candidates take USMLE as a component of their application. USMLE scores are reported as means and as categorical groups of 10. Depending on the near-mean and specialty competitiveness strata, Step 1 scores were stratified into two-level contingencies with a cut point of 230 and Step 2 at a cut point of 240 or 250. The NRMP does not directly report the number of completed interviews, but rather reports how many programs a candidate ranks in a particular specialty, which serves as an adequate variable proxy for our purposes. A three-level contingency was created with three groups (<6, 6-15, and >15 programs ranked) for comparison.

Data analysis and storage were performed using Prism 9 (GraphPad Software, San Diego, CA, USA). Descriptive data are reported as mean ± standard deviations if continuous, counts if categorical, or as simple proportions. Variable thresholds were set for categorical analysis for NLEs, research output, and programs ranked. Analysis was conducted via the Chi-square test or Fisher exact test if counts were less than five. A p-value of 0.05 determined significance with reported odds ratios (OR) utilizing a confidence interval of 95%.

## Results

There were 46,853 total US MD and DO Senior candidates available for analysis in the 2018 and 2020 COM publications. After competitiveness stratification, there were 20,570 LC (43.9%), 21,171 MC (45.2%), and 5,112 HC (10.9%) candidates. Considering applicant type, there were 5,846 US DO Seniors in the LC cohort (28.4%), 4,417 in the MC cohort (20.9%), and 396 in the HC cohort (7.8%), with US MD Seniors comprising the remainder.

Match performance 

**Figure 1:**
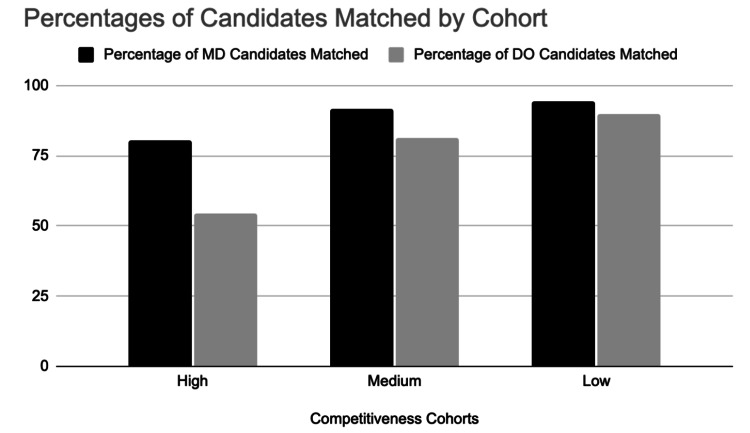
Match performance among US MD and DO Seniors stratified by specialty competitiveness. MD, Doctor of Medicine; DO, Doctor of Osteopathic Medicine

Match rates for US MD Seniors were significantly higher than US DO Seniors for all groups: US DO LC = 89.92%, US MD LC = 94.36% (OR = 1.90, p < 0.0001), US DO MC = 81.50%, US MD MC = 91.45% (OR = 2.43, p < 0.0001), and US DO HC = 54.29%, US MD HC = 80.56% (OR = 3.49, p < 0.0001) (Figure 1). When considering specific specialties, match rates in Pathology did not differ significantly between the US MD and DO Senior groups (p = 0.25), while all other individual specialties analyzed for which adequate data was available were significantly different (p < 0.05).

Research output

Matched US MD Seniors were significantly more likely to report greater (≥5 vs <5) numbers of research experiences in the LC cohort (OR = 2.23, p < 0.0001), MC cohort (OR = 2.36, p < 0.0001), and HC cohort (OR = 4.09, p < 0.0001). Matched US MD Seniors were significantly more likely to report greater (≥5 vs <5) numbers of research items in the LC cohort (OR = 2.37, p < 0.0001) and HC cohort (OR = 3.96, p < 0.0001), but not in the MC cohort (OR = 1.02, p = 0.76) (Table 2).

**Table 2:** Mean research output of matched US MD and DO Seniors stratified by specialty competitiveness. LC, low competitiveness; MC, moderate competitiveness; HC, high competitiveness; US, United States; MD, Doctor of Medicine; DO, Doctor of Osteopathic Medicine

Research experiences	Cohort	US MD Seniors (n=36,194)	US DO Seniors (n=10,659)
	LC	3.0	1.9
	MC	3.3	2.1
	HC	5.3	3.9
Research items			
	LC	5.5	2.8
	MC	5.4	2.9
	HC	13.9	11.6

USMLE performance

The US MD Seniors were significantly more likely than US DO Seniors to score >230 on the USMLE Step 1 examination in the LC cohort (OR = 1.87, p < 0.0001) and MC cohort (OR = 1.43, p < 0.0001). The US MD Seniors were more likely than US DO Seniors to score >250 on the USMLE Step 1 examination in the HC cohort (OR = 1.64, p = 0.002). The US MD Seniors were significantly more likely than US DO Seniors to score >240 on the USMLE Step 2 CK examination in the LC cohort (OR = 2.00, p < 0.0001) and MC cohort (OR = 1.75, p < 0.0001). The US MD Seniors were more likely than US DO Seniors to score >250 on the USMLE Step 2 CK examination in the HC cohort (OR = 1.58, p = 0.005) (Table 3).

**Table 3:** Mean USMLE Step 1 and Step 2 CK scores of matched US MD and DO Seniors stratified by specialty competitiveness. USMLE, United States Medical Licensing Examination; CK, clinical knowledge; MD, Doctor of Medicine; DO, Doctor of Osteopathic Medicine; LC, low competitiveness; MC, moderate competitiveness; HC, high competitiveness; US, United States

USMLE Step 1 Score	Cohort	US MD Seniors (n=36,194)	US DO Seniors (n=10,659)
	LC	229.5	222.8
	MC	232.7	228.7
	HC	247.6	244.7
USMLE Step 2 CK Score			
	LC	243.3	236.6
	MC	247.4	241.1
	HC	254.5	250.2

Contiguous programs ranked

Matched US MD Seniors ranked significantly more programs than their US DO Senior counterparts and matched US DO Seniors were more likely to rank fewer than five programs in their preferred specialty. The US MD Seniors were significantly more likely to rank more programs in all competitiveness strata (p < 0.0001) (Tables 4-5).

**Table 4:** Mean number of contiguous programs ranked by matched US MD and DO Seniors stratified by specialty competitiveness. LC, low competitiveness; MC, moderate competitiveness; HC, high competitiveness; MD, Doctor of Medicine; DO, Doctor of Osteopathic Medicine; US, United States

Cohort	US MD Seniors (n=36,194)	US DO Seniors (n=10,659)
LC	11.9	10.3
MC	12.7	9.8
HC	12.5	7.2

**Table 5:** Proportion of matched US MD and DO Seniors ranking certain numbers of programs in their preferred specialty stratified by specialty competitiveness. LC, low competitiveness; MC, moderate competitiveness; HC, high competitiveness; MD, Doctor of Medicine; DO, Doctor of Osteopathic Medicine; US, United States

		US MD Seniors (n=36,194)			US DO Seniors (n=10,659)	
Cohort	LC	MC	HC	LC	MC	HC
≤5 Programs	1192	918	598	821	541	98
	9.3%	9.3%	15.5%	16.9%	16.1%	48%
6-15 Programs	8729	9662	2203	3382	2402	99
	68.2%	66.4%	57.3%	69.6%	71.5%	48.5%
≥16 Programs	2873	3970	1047	659	416	7
	22.5%	27.3%	27.2%	13.6%	12.4%	3.4%

## Discussion

In this study, we aim to characterize and compare the NRMP performance of US MD and DO Seniors in the early single accreditation era. The US MD Seniors observed better match performance than US DO Seniors across all competitiveness strata, which may be due to several factors. One of the primary differences between US MD and DO applicant profiles relates to research involvement and output. The US MD Seniors had more research products and experiences compared to US DO Seniors in most specialties. Interestingly, there was no observed difference in the MC cohort between the groups, which may represent varying degrees of importance for research involvement between surgical and non-surgical specialties [14]. Nevertheless, research is theoretically important for all candidates to demonstrate creative and scientific thinking, a willingness to engage with changing aspects of the field, and the ability to accommodate paraclinical duties in the face of clinical requirements [18].

Research involvement and attendance at institutions with more National Institutes of Health (NIH) funding are known to be independent predictors of match success [11, 19]. Research may have previously been more important and scholarly engagement more greatly emphasized for allopathic candidates, but this could change in the single accreditation GME system with more US DO Seniors conducting and publishing research [11-20]. A recent survey found that nearly half of osteopathic graduates perceived inadequate resources dedicated to research technique development, literature analysis, and biostatistics [21]. Additionally, osteopathic physicians and medical schools produce less research and obtain less NIH funding than their allopathic counterparts [13, 20, 22-23]. Due to these differences, osteopathic medical students must work harder to identify research mentors and develop projects in their desired field. This could potentially disrupt osteopathic medical students’ ability to adequately focus on academic and clinical work or delay their graduation to complete research fellowships, contributing to an increased financial burden.

Second, matched US MD Seniors were more likely to score higher on the USMLE compared to US DO Seniors across all cohorts. Four things must first be considered: (1) the NLE landscape for future US physicians is rapidly changing, (2) many, but not all, US DO Seniors take USMLE Step 1 and/or 2 CK, (3) many confounders can explain score discrepancies such as preferred specialty, qualitative factors like resilience, and demographics, and (4) NLEs are not written for the purpose of residency candidate selection. Since some US DO Seniors do not take USMLE Step 1 and elect to only take the COMLEX examinations, this data should not be generalized to all students who applied but rather be used as an indication of the competitiveness of those students who sat for USMLE Step 1. Conversions do exist for COMLEX to USMLE score interpretations; however, these have been proven to be inconclusive and not representative [24]. Despite these priors, USMLE scores may indicate dedication, academic prowess, and available resources to achieve one’s goals [25]. In any case, this may be explained by several factors. First, osteopathic medical schools may not emphasize taking or preparing for the USMLE and thus provide USMLE-specific study resources for their students, which could lead to an increased financial burden placed on the student. This likely discourages many from taking USMLE or could make preparation for it more cumbersome. Second, exam fatigue may hinder a DO student's ability to optimize their performance on either USMLE or COMLEX. If US DO Seniors take both exams in the same week, this could potentially negatively impact test performance. Finally, US MD Seniors on average may emphasize NLE preparation to a greater degree given that proportionately more apply to HC specialties than US DO Seniors, resulting in better performance on standardized examinations. Nevertheless, given that USMLE Step 1 and COMLEX Level 1 will move to pass/fail grading in 2022, the impetus to improve other application areas may grow, and students from less prestigious schools may face disproportionate challenges [10, 26]. It is discussed that NLEs may not capture the qualities necessary to assess residency training candidacy so more holistic means to evaluate applicants have been conceptualized [10, 27]. Until changes to the ERAS and NRMP are made, residency programs will likely continue to rely on NLEs in candidate selection.

Finally, while not a direct representation, the number of programs an applicant ranks in ERAS serves as an adequate proxy for the number of interviews offered and subsequently completed. Matched US MD Seniors ranked significantly more programs than their US DO Senior counterparts. This is especially true in the HC cohort, where US MD Seniors were about nine-fold more likely to rank greater than 15 programs in ERAS than US DO Seniors. This may be related to the aforementioned factors such as NLE performance and scholarly output, but in this new environment of single GME training, it is concerning that osteopathic candidates rank fewer programs, and thus be extended fewer interview offers, than allopathic candidates. While certainly not ubiquitous, bias against osteopathic candidates may remain, as suggested by the NRMP’s Program Director Survey (PDS). In 2020, for all specialties, 36% of program directors reported that they will “seldom” or “never” interview US DO Seniors compared to 6% for US MD Seniors [28]. Again, the discrepancy is greater in highly competitive specialties. For example, in dermatology, 92% of program directors reported “seldom” or “never” interviewing US DO Seniors while 100% stated they interview US MD Seniors [28]. Indeed, for most specialties, the PDS response rate is low, and these specialties also do not receive many osteopathic applications consistently every year. Additionally, the average US DO Senior applicant may have fewer professional connections within their preferred specialty, secondary to not having a home residency program. It may also be that competitive programs and specialties fear how accepting otherwise qualified DO candidates from lesser-known institutions are perceived with regard to prestige. Whatever the reason, given that osteopathic students now represent about a quarter of all medical students in the US, competitive specialties may see a greater number of osteopathic applicants [22]. Time will elucidate the true level of bias among specialties more broadly in the single accreditation GME system. 

Study limitations

The lack of data transparency limits our ability to conduct quantitative analysis of individual applicants, which makes our study susceptible to confounding bias. In 2018, the GME partnership had not yet been completed, so we could not account for the applicants who may have been accepted into AOA programs, particularly in HC specialties. This could thus alter the data for osteopathic applicant profiles and acceptance rates, subjecting the study to sampling bias. This data combination (2018 and 2020 data) was performed after considering the risk for bias so as to provide greater numbers of applicants for more conclusive statistical significance due to the low DO application rates in some specialties. Finally, we pooled candidates into competitiveness groups and used programs ranked as a proxy for interview offers, subjecting the study to design bias. This organization was performed in concordance with previous studies to follow precedent and allow longitudinal comparisons across time [7, 11].

Future perspective

There are several other variables likely related to match discrepancies among US MD and US DO Seniors that we did not investigate but should be considered, including the impact of mentorship, applicant demographics, socioeconomic factors, student access to affiliated hospital systems and in-house residency programs, quality of letters of recommendation, extracurriculars, involvement with national specialty organizations, and how the COVID-19 pandemic affected applicants in the 2020 NRMP [8-9]. These may become even more relevant with the upcoming structural changes to USMLE Step 1 and COMLEX Level 1, as well as the annulment and/or restructuring of the USMLE Step 2 CS (Clinical Skills) and COMLEX Level 2 PE (Performance Evaluation) examinations. Future studies will be needed to establish new US MD and DO Senior comparisons after 2024 when students who are affected by all these changes will graduate. Future research may additionally be directed towards the impact of the limited scholarly opportunities available to US DO applicants on the resident’s ability to perform and interpret research.

Matching into a residency position is complicated. Given the financial burden and emotional stress associated with the process, more robust and honest conversations about applicants’ candidacy can prove to be beneficial [29]. Although US DO Seniors have observed anecdotally encouraging trends in overall match performance since the single accreditation GME system went into effect, US DO Senior status appears to be an overall independent predictor of the poorer match performance. We have uniquely observed several significant discrepancies between these cohorts, especially for highly competitive specialties, in applicant research involvement, NLE performance, and numbers of programs ranked. Addressing these and other variables not analyzed will take creativity and new resource allocation strategies among individual applicants, medical schools, and accrediting bodies. Osteopathic medical schools in particular will need to continually evaluate the needs of their students and provide resources and training beyond current accreditation standards.

## Conclusions

Matching into residency training in the United States is competitive. After the single accreditation GME system between the ACGME, AACOM, and AOA finalized in 2020, we aimed to characterize and compare the early match performance trends among US MD and DO Seniors, stratified by specialty competitiveness. The US MD Seniors had higher match rates than US DO Seniors across all strata, with the greatest difference observed in highly competitive specialties. To explain this difference, US DO Seniors also observed lower research output, NLE scores, and number of programs ranked compared to US MD Seniors to varying degrees. While other factors likely contribute, osteopathic medical students and their institutions need to focus more resources on research, advising, and board preparation to improve their candidacy for future NRMPs.
